# Factors that influence mothers’ prenatal decision to breastfeed in Spain

**DOI:** 10.1186/s13006-020-00341-5

**Published:** 2020-11-17

**Authors:** Ana Ballesta-Castillejos, Juan Gómez-Salgado, Julián Rodríguez-Almagro, Inmaculada Ortiz-Esquinas, Antonio Hernández-Martínez

**Affiliations:** 1 Department of Obstetrics & Gynaecology, Centro de Salud San Clemente, Cuenca, Spain; 2 Department of Sociology, Social Work and Public Health, University of Huelva, 21071 Huelva, Spain; 3 Safety and Health Postgrade Program, Universidad Espíritu Santo, 091650 Guayaquil, Ecuador; 4 Department of Nursing, Faculty of Nursing of Ciudad Real, University of Castilla-La Mancha, Ciudad Real, Spain; 5 Department of Obstetrics & Gynaecology, Complejo Hospitalario Jaén, Jaén, Spain

**Keywords:** Breastfeeding, Partner support, Decision to breastfeed, Internet, Midwife

## Abstract

**Background:**

Parents’ decisions about how to feed their newborns are influenced by multiple factors. Our objective was to identify the factors that can influence the decision to breastfeed.

**Methods:**

Cross-sectional observational online study was conducted in Spain on women who gave birth between 2013 and 2018. The total number of participants was 5671. Data collection was after approval by the ethics committee in 2019. The data were collected retrospectively because the information was obtained from women who were mothers during the years 2013–2018. An online survey was distributed to breastfeeding associations and postpartum groups. Multivariate analysis with binary logistic regression was done to calculate the Adjusted Odds Ratios (aOR). The main result variable was “intention to breastfeed”.

**Results:**

Ninety-seven percent (*n* = 5531) of women made the decision to breastfeed prior to giving birth. The internet played a role in deciding to breastfeed in 33.7% (*n* = 2047) of women, while 20.1% (*n* = 1110) said the same thing about their midwife. We identified five significant factors associated with the mother’s prenatal decision to breastfeed: attending maternal education (aOR 2.10; 95% CI 1.32, 3.34), having two (aOR 0.52; 95% CI 0.28, 0.99) and three children (aOR 0.24; 95% CI 0.10, 0.59), previous breastfeeding experience (aOR 6.99; 95% CI 3.46, 14.10), support from partner (aOR 1.58; 95% CI 1.09,2.28) and having a condition during pregnancy (aOR 0.62; 95% CI 0.43, 0.91).

**Conclusions:**

Factors related with previous breastfeeding experience and education for mothers are decisive when it comes to making the decision to breastfeed. Given the proven influence that partners have in decision-making, it is important for them to be fully involved in the process.

**Supplementary Information:**

The online version contains supplementary material available at 10.1186/s13006-020-00341-5.

## Background

The World Health Organization (WHO) recommends that all babies receive exclusive breastfeeding (EBF) during the first 6 months of life and to continue with breastfeeding along with complementary foods until at least the age of two [1]. However, rates for exclusive breastfeeding continue to be low worldwide, only 36% of babies under 6 months receive EBF [1, 2]. In Spain, there is no official system suitable for monitoring breastfeeding [3] but data extracted from National Health Surveys show that the rate of breastfeed in Spain (including EBF and partial breastfeeding) at 6 weeks has remained quite stable since 1995, with overall figures of around 71%. Breastfeeding rates at 3 and 6 months have progressively increased in the past 5 years, reaching 66.5 and 46.9%, respectively, which is an improvement but still far from the WHO recommendations [3].

An increasing number of studies suggest that parents are influenced by multiple sociocultural factors that interact to guide their infant feeding decision [4]. It has been suggested that decision-making on infant feeding begins before pregnancy and is finalised in the prenatal period. In fact, studies have identified that prenatal breastfeeding intentions are closely related with real feeding practices [4].

Previous international studies have shown a connection between the following factors and the mother’s intention to exclusively breastfeed: positive attitudes toward EBF, perceived social support and monitoring of behaviour, previous experience with exclusive breastfeeding, the mother being breastfed as a baby, older maternal age, a high level of education and knowledge of the benefits of exclusive breastfeeding [2, 5, 6]. However, evidence in this field is lacking, especially in relation to the population resident in Spain.

Based on the available evidence, it seems clear that we need to find out which factors influence the decision to breastfeed in order to take steps to increase breastfeed initiation and duration rates, therefore, the objective is to identify the factors that can influence the decision to breastfeed.

## Methods

### Design

Cross-sectional observational study in Spain.

### Setting

Study carried out in Spain on women who have been mothers between 2013 and 2018. Data were collected between 2018 and 2019. For sample recruitment, the breastfeeding associations from the different Spanish provinces were contacted via email, and these were in charge of disseminating the questionnaire to all their members through email.

### Sample

This was a convenience sample study design. To participate in the study, women aged 19 or older, who were mothers to children between 0 and 5 years of age, understood Spanish, and agreed to participate in the study by completing an online questionnaire were selected.

To estimate the sample size, the criterion of maximum modelling was used, which requires 10 events (prenatal intention to formula feed) for each independent variable to include in the multivariate model [7]. For a minimum of 20 independent variables, we would need a minimum of 200 women with a prenatal intention not to breastfeed. Due to the fact that prevalence of the prenatal intention to formula feed stood at 14.7% in previous studies [5], a minimum of 1340 women would be needed. Despite this estimate, the research team decided to enlist a greater number of women as the prenatal intention to breastfeed in our population was unknown.

### Data collection

To collect the data, an anonymous online questionnaire was distributed to breastfeeding associations and postpartum groups (Additional file 1: List of associations that have participated in the distribution of the questionnaire). Women with children aged 0 to 5 were invited to participate. The questionnaire was filled in after birth. Before completing the questionnaire, the participants had to read the information on the purpose of the study and give their consent to take part. They were then given the information necessary to respond to the questionnaire. Voluntarily, participants could leave an email address or telephone number to be contacted if any additional information was needed in relation to the study.

### Measurement

The questionnaire was composed of 22 items (5 yes/no questions, 16 Likert-type questions and 1 open-response question) about socio-demographic variables, obstetric variables, influences on the decision to breastfeed and motivations for deciding on the type of breastfeeding.

The variables included in the study were:

The main dependent variable: mother’s prenatal decision to breastfeed (yes/no).

The independent variables were:

Socio-demographic factors: maternal age, employment status, income, level of education, nationality, partner support, family support, professional support.

Obstetric factors: Body Mass Index (BMI), attended antenatal education, tobacco used before pregnancy, manner of conception, number of children, twin pregnancy, previous caesarean, and previous children.

Complications during pregnancy: hypertension states, gestational diabetes diet, gestational diabetes insulin, hyperthyroidism, hypothyroidism, anaemia, intrahepatic cholestasis, risk of premature birth, deep vein thrombosis, oligohydramnios, polyhydramnios and composite morbidity pregnancy as grouping variable of all the pathologies during pregnancy. The women were asked to mark the existence of a pathology only after having been diagnosed by a physician.

The variable “external influences” is a variable made up of the sum of the scores of 8 variables (influence of friends, family members, internet, midwife, nurses, primary care physicians, paediatrician and gynaecologist) where women were asked to rate, on a scale from 0 (not influential) to 3 (very influential), the degree of influence of each of these concepts on their decision-making Table 3.

The variable “main reason for breastfeeding” was, in turn, made up of 8 reasons, where women were asked to rate the degree of influence on a scale from 0 (not influential) to 3 (very influential). e.g. “I think it’s the best way of feeding my baby”, “It’s good for my baby’s health”, “Due to the benefits for the mother”) Table 4.

### Data analysis

The data were analysed descriptively using absolute and relative frequencies for categorical variables. Next, the chi-squared analysis was done between the socio-demographic and clinical variables and the mother’s prenatal decision to breastfeed. Then, multivariate analysis was done using binary logistic regression using the forward and backward stepwise regression procedures in SPSS. In this multivariate analysis, all the independent variables were initially included, but only those showing statistical relationship with the main result variable were eventually included in the final model. For those variables that showed statistical significance, adjusted odd ratios (aOR) were calculated with confidence intervals of 95% (CI 95%), as well as the area under the ROC curve of the final model with their respective CI of 95%.

Finally, an indicator was created to estimate the overall influence of external persons and the overall influence of the motivations for breastfeeding. This indicator takes a value of between 0 and 100 and estimates the individual effect of each influence or reason out of the total set of influences and motivations. It was calculated using the average of the quotient (score for each influence or reason / total scores for all influences or reasons) × 100. In addition, Cronbach’s Alpha was determined both for the “external influences” variable and the “main reason for breastfeeding” variable. All analyses were done using the SPSS v24.0 statistics package.

## Results

The study population was 5671 women. Of these, 97.5% (*n* = 5531) made the decision to breastfeed before giving birth. Of the 5531 women who decided to breastfeed, 81.1% (4485) could apply exclusive maternal breastfeeding at discharge. Next, bivariate analysis was done to determine the factors associated with the intention to breastfeed. In relation to the socio-demographic and obstetric factors (Table 1), the intention to breastfeed was found to be statistically related with maternal age (*p* = 0.043), the mother’s nationality (*p* = 0.024), attending maternal education (*p* = 0.002), number of children (*p* = 0.003), twin pregnancy (*p* = 0.036), breastfeeding with previous children (*p* <  0.001), the mother’s perception of her partner supporting her in her decision to breastfeed (*p* = 0.003) and the mother’s perception of professionals supporting her in her decision to breastfeed (*p* = 0.018). As for the relationship between the intention to breastfeed and pregnancy complications, the only statistically significant associations observed were with composite morbidity (a variable made up of all complications during pregnancy) (*p* = 0.010).

**Table 1:** Socio-demographic and obstetric characteristics of women by mother’s prenatal decision to breastfeed

Variable	Decision to breastfeed		
	No ***n*** (%)	Yes ***n*** (%)	***P*** - value^a^
**Maternal Age**			**0.043**
≤ 20 years	1 (9.1)	10 (90.9)	
21–30 years	33 (3.5)	910 (96.5)	
31–40 years	101 (2.3)	4269 (97.7)	
> 40 years	5 (1.4)	342 (98.6)	
**Employment status**			0.567
Doesn’t work	57 (2.5)	2232 (97.5)	
Works part-time	30 (2.1)	1385 (97.9)	
Works full-time	53 (2.7)	1914 (97.3)	
**Income**			0.555
< 1000 euros	8 (2.5)	307 (97.5)	
1000–2000 euros	44 (2.4)	1820 (97.6)	
2000–3000 euros	54 (2.8)	1899 (97.2)	
3000–4000 euros	20 (1.9)	1054 (98.1)	
> 4000 euros	14 (3.0)	451 (97.0)	
**Level of education**			0.091
No formal education	1 (12.5)	7 (87.5)	
Primary education	5 (5.1)	93 (94.9)	
Secondary education	39 (2.6)	1480 (97.4)	
University education	95 (2.3)	3951 (97.7)	
**Nationality**			**0.024**
Spanish	125 (2.5)	5195 (97.7)	
Other	15 (4.3)	336 (95.7)	
**BMI before pregnancy**			0.650
Normal (18.5–24.9)	35 (2.3)	1517 (97.7)	
Overweight (25–29.9)	61 (2.3)	2543 (97.7)	
Obese (≥ 30)	40 (2.7)	1422 (97.3)	
**Attended maternal prenatal education**			**0.002**
No	35 (2.8)	1235 (97.2)	
Yes, but not about breastfeeding	40 (3.8)	1007 (96.2)	
Yes	65 (1.9)	3289 (98.1)	
**Tobacco use before pregnancy**			0.133
No	104 (2.4)	4258 (97.6)	
1–10 cigarettes	22 (2.3)	945 (97.7)	
> 10 cigarettes	14 (4.1)	328 (95.9)	
**Manner of conception**			0.611
Spontaneous	124 (2.4)	4962 (97.6)	
Insemination/ IVF^b^	16 (2.7)	569 (97.3)	
**Number of children**			**0.003**
One	104 (2.9)	3440 (97.1)	
Two	27 (1.5)	1827 (98.5)	
Three and more	9 (3.3)	264 (96.7)	
**Twin Pregnancy**			**0.036**
No	133 (2.4)	5405 (97.6)	
Yes	7 (5.3)	126 (94.7)	
**Previous Caesarean**			0.527
No	129 (2.5)	5009 (97.5)	
Yes	11 (2.1)	522 (97.9)	
**Breastfeed previous children**			**< 0.001**
No	116 (3.2)	3503 (96.8)	
Yes	24 (1.2)	2028 (98.8)	
**Partner support**			**0.003**
No	42 (3.7)	1098 (96.3)	
Yes	98 (2.2)	4433 (97.8)	
**Family support**			0.053
No	97 (2.8)	3386 (97.2)	
Yes	43 (2.0)	2145 (98.0)	
**Professional support**			**0.018**
No	68 (3.1)	2137 (96.9)	
Yes	72 (2.1)	3390 (97.9)	
**Composite morbidity pregnancy**			**0.010**
No	38 (1.8)	2093 (98.2)	
Yes	102 (2.9)	3438 (97.1)	

^a^ Pearson’s χ^2^ test

^b^ IVF means In Vitro Fertilization

In the next step, multivariate analysis was done using binary logistic regression, incorporating all of the variables that could potentially be related with the intention to breastfeed (statistical criteria *p* ≤ 0.20). Following this analysis (Table 2), we observed five factors associated with the mother’s prenatal decision to breastfeed. Thus, women who had attended maternal education with breastfeeding training were twice more likely to breastfeed (aOR 2.10, CI 95% 1.32, 3.34), as compared to those that had not; women with two or three or more children were 0.52 and 0.24 time less likely to breastfeed than women with one child; those who had breastfed previous children were almost seven times more likely to have the intention to breastfeed (aOR 6.99, CI 95% 3.46, 14.10), as compared to those who had not; those who had support from their partner were 1.5 times more likely to breastfeed aOR 1.58, CI 95% 1.09, 2.28), as compared to those who did not receive this support; and women with a condition during pregnancy (composite morbidity) were 0.62 times less likely to breastfeed (aOR 0.62, CI 95% 0.43, 0.91), as compared to those who did not suffer it. The AUC-ROC for this model was 0.70 (CI 95% 0.65, 0.74).

**Table 2:** Factors associated with the intention to breastfeed after performing the multivariate analysis

Variable	Decision to breastfeed		
	aOR	CI 95%	***P*** - value*
**Attended maternal prenatal education**			
No	1		
Yes, but I didn’t attend training on breastfeeding	1.05	(0.64, 1.17)	0.847
Yes	**2.10**	**(1.32, 3.34)**	**0.002**
**Number of children**			
One	1		
Two	**0.52**	**(0.28, 0.99)**	**0.045**
Three and more	**0.24**	**(0.10, 0.59)**	**0.002**
**Breastfeed in the past**			
No	1		
Yes	**6.99**	**(3.46, 4.10)**	**< 0.001**
**Partner support**			
No	1		
Yes	**1.58**	**(1.09, 2.28)**	**0.016**
**Composite morbidity pregnancy**			
No	1		
Yes	**0.62**	**(0.43, 0.91)**	**0.015**

*aOR* Odds Ratio values adjusted by all the variables included in this model after performing the multivariate analysis

*p - value obtained after adjusting by the rest of variables included in the model

Moreover, mothers were also asked to score the degree of influence that several external persons had had on them (midwives, nursing professionals, gynaecologists, paediatricians, family doctors, friends and social media/internet), as well as the motivations that led them to make the prenatal decision to breastfeed. Cronbach’s alpha for these variables was 0.80.

In the first case, the highest average score was for social media/internet with 1.90 (SD = 1.07), followed by the role of the midwife with an average score of 1.41 (SD = 1.11). The estimated overall influence of the total set of external influences was, in the case of social media/internet, 33.85%, while for the role of the midwife the overall influence was 20.2% of the overall influence (scale of 0–100%). The information for the rest of the external persons is set out in detail in Table 3.

**Table 3:** Influence of external persons on the decision to breastfeed in women who had decided to breastfeed before giving birth

Factor	***n*** (%)	Mean (SD)	Median (IQR)	Mean Overall influence^a^ (SD)
**Midwives**		1.41 (1.11)	1 (2)	20.2 (18.20)
Not influential	1602 (29.0)			
Not very influential	1165 (21.1)			
Quite influential	1654 (29.9)			
Very influential	1110 (20.1)			
Missing values	0			
**Nurses**		0.50 (0.86)	0 (1)	4.68 (7.99)
Not influential	3823 (69.1)			
Not very influential	904 (16.3)			
Quite influential	524 (9.5)			
Very influential	280 (5.1)			
Missing values	0			
**Gynaecologists**		0.51 (0.85)	0 (1)	4.68 (7.99)
Not influential	3741 (67.7)			
Not very influential	1000 (18.1)			
Quite influential	529 (9.6)			
Very influential	261 (4.7)			
Missing values	0			
**Paediatricians**		0.75 (1.00)	0 (1)	7.86 (10.90)
Not influential	3176 (57.4)			
Not very influential	1062 (19.2)			
Quite influential	815 (14.7)			
Very influential	478 (8.6)			
Missing values	0			
**Family doctors**		0.38 (0.74)	0 (0)	3.10 (5.69)
Not influential	4153 (75.1)			
Not very influential	837 (15.1)			
Quite influential	379 (6.9)			
Very influential	162 (2.9)			
Missing values	0			
**Family**		1.00 (1.02)	1 (2)	14.20 (17.43)
Not influential	2331 (42.1)			
Not very influential	1420 (25.7)			
Quite influential	1234 (22.3)			
Very influential	546 (9.9)			
Missing values	0			
**Friends**		0.89 (0.99)	1 (2)	11.31 (13.67)
Not influential	2624 (47.4)			
Not very influential	1374 (24.8)			
Quite influential	1060 (19.2)			
Very influential	473 (8.6)			
Missing values	0			
**Internet/social media**		1.90 (1.07)	2 (2)	33.85 (26.40)
Not influential	894 (16.2)			
Not very influential	806 (14.6)			
Quite influential	1784 (32.3)			
Very influential	2047 (37.0)			
Missing values	0			

*SD* Standard deviation, *IQR* Interquartile range

^a^This value is the average score of (influence of each factor / total scores of all influences) × 100. With this indicator, the total influence of all factors is 100

In the second case, the highest average score was for the reason “It’s good for my baby’s health” with an average score of 2.78 (SD = 0.46) followed by the reason “I think it’s the best way of feeding my baby” with 2.77 (SD = 0.46). The estimated overall influence of the total set of motivations was 20.44% for the first reason and 20.37% for the second reason (scale of 0–100%). Cronbach’s alpha for these variables was 0.74.

The information for the rest of the motivations is set out in detail in Table 4.

**Table 4:** Influence of the reasons to breastfeed on women

Factor	***n*** (%)	Mean (SD)	Median (IQR)	Mean Overall influence^a^ (SD)
**I think it’s the best way of feeding my baby.**		2.77 (0.46)	3 (3)	20.37 (5.90)
Not influential	17 (0.3)			
Not very influential	47 (0.8)			
Quite influential	1087 (19.7)			
Very influential	4215 (76.2)			
Missing values	165 (3.0)			
**It’s good for my baby’s health.**		2.78 (0.46)	3 (3)	20.44 (6.12)
Not influential	17 (0.3)			
Not very influential	36 (0.7)			
Quite influential	1075 (76.0)			
Very influential	4201 (96.3)			
Missing values	202 (3.7)			
**Recommendation by midwife or nurse.**		1.36 (1.06)	3 (2)	8.73 (6.52)
Not influential	1407 (25.4)			
Not very influential	1496 (27.0)			
Quite influential	1433 (25.9)			
Very influential	929 (16.8)			
Missing values	266 (4.8)			
**Because I didn’t consider any other alternatives.**		1.69 (1.20)	3 (3)	10.91 (7.86)
Not influential	1325 (24.0)			
Not very influential	911 (16.5)			
Quite influential	1129 (20.4)			
Very influential	1908 (34.5)			
Missing values	258 (4.7)			
**It is an intimate moment with my baby.**		2.38 (0.83)	3 (1)	16.57 (5.68)
Not influential	210 (3.8)			
Not very influential	544 (9.8)			
Quite influential	1551 (28.0)			
Very influential	2990 (54.1)			
Missing values	236 (4.3)			
**Recommendation from gynaecologist.**		0.53 (0.83)	3 (1)	3.02 (4.52)
Not influential	3395 (61.4)			
Not very influential	1142 (20.6)			
Quite influential	491 (8.9)			
Very influential	220 (4.0)			
Missing values	283 (5.1)			
**Due to the benefits for the mother.**		2.03 (0.99)	3 (2)	13.75 (6.49)
Not influential	499 (9.0)			
Not very influential	1007 (18.2)			
Quite influential	1602 (29.0)			
Very influential	2188 (39.6)			
Missing values	235 (4.2)			
**Due to the support received and experiences of childbirth.**		1.03 (1.07)	3 (2)	6.19 (6.18)
Not influential	2235 (40.4)			
Not very influential	1382 (25.0)			
Quite influential	926 (16.7)			
Very influential	731 (13.2)			
Missing values	257 (4.6)			

*SD* Standard deviation, *IQR* Interquartile range

^a^ This value is the average score of (reasons for each factor / total scores of all reasons) × 100. With this indicator, the reasons total 100

## Discussion

Adequate nutrition during infancy and early childhood is essential to ensure that children reach their full potential in terms of growth, health and development [1]. Early nutritional deficiencies have been linked to problems that compromise growth and health in the long term [1]. The demonstrated benefits of breastfeed and its superiority over formula feeding (FF) are now undisputed and are clear from the WHO and UNICEF recommendations for optimal infant feeding, which is set out in the Global Strategy, to include exclusive breastfeeding for the first 6 months of life (180 days) and the introduction of adequate and safe complementary foods from 6 months, with continued breastfeeding up to 2 years or beyond [1, 8, 9].

The majority of women make the decision in the first trimester or even before becoming pregnant or before any contact with maternal and child services, although some make the decision at the end of pregnancy or even after giving birth [10]. The intention to breastfeed is a determining factor when it comes to initiating it, especially in relation to the duration of exclusive breastfeed [11, 12]. The process of beginning and maintaining breastfeed is very vulnerable to external influences and social factors [13, 14] which means that many women who wish to breastfeed may fail to do so [10].

In this study, five factors were identified associated with the mother’s prenatal decision to breastfeed: partner support, previous experience of breastfeeding, having two or more children, attending breastfeeding education, and having a condition during pregnancy.

In line with the conclusions of other research [15, 16], having a partner with a favourable attitude toward breastfeed may be a decisive factor when it comes to making the decision to breastfeed and may even determine its duration, as the partner is the mother’s main support during breastfeeding [17]. This may reflect the intense influence that close relatives can exert on the mother’s decision whether or not to breastfeed [5] which can even be greater than that exerted by the healthcare team [15, 18].

Previous breastfeeding experience has also been identified as a factor associated with the mother’s prenatal decision to breastfeed, as we have confirmed in our study, and there is scientific evidence that mothers with a positive breastfeeding experience with a previous child are more likely to breastfeed with subsequent children [2, 15, 18, 19].

Currently, the importance of maternal education classes is undisputed and most pregnant women in our setting have a referral centre where they can access these [20]. There are studies that suggest that maternal education given by midwives from the first antenatal visit is a protective factor for initiating and maintaining breastfeeding [10, 21]. In this study, we explored this topic further by dividing women into three groups: women that did not attend maternal education, women that attended maternal prenatal education but did not receive information on breastfeeding, and women that attended training and received information on breastfeeding. This shows that the inclusion of information on breastfeeding in maternal education has a positive effect on women’s decision to breastfeed. These data are consistent with those obtained in studies such as Lutsiv et al. [22] which found greater rates of formula feeding in mothers who did not attend maternal prenatal education, with some studies recommending attending maternal education to promote breastfeeding [2].

In this study, it has been identified that having existing children is a factor that is negatively associated with the mother’s prenatal decision to breastfeed, in the same line as the results observed by Lee et al. [23]. This could indicate that the mother’s previous experiences may act as positive reinforcement if the previous experiences were positive, but could also become negative reinforcement if the previous experiences were negative. In this regard, we can also consider that the increase in family responsibilities due to the greater number of children in the home could pose a challenge for breastfeeding, so it may be necessary to implement support strategies aimed at this population. In this regard, we think that the partner should take on greater responsibility and, as far as possible, release the woman from her usual household tasks so she can concentrate on recovery and on breastfeeding.

Women with conditions during pregnancy such as hypertension or diabetes are less likely to breastfeed than those who do not suffer these conditions. Thus, the implications that conditions during pregnancy may play a relevant role in breastfeeding [24, 25]. Our results are also in line with this, as conditions during pregnancy have been identified as a decisive factor when it comes to decision-making about whether to breastfeed.

It is undisputed that the family and the healthcare team play an important role when it comes to the decision to breastfeed, however, our analysis shows that the most significant external influence is the internet/social media. This contrasts with another studied done by Díaz-Gómez in 2016 in which the family is identified as the main external influence when it comes to making the decision to breastfeed [26] and now indicates how new technologies are taking on an increasingly important role in all aspects of our lives. Recently, several studies have been published on the influence of certain online actions to support breastfeeding, demonstrating positive effects on its duration [27, 28]. For this reason, we agree with other authors in [29] that the combination of online actions and more traditional methods such as maternal education led by health professionals, namely by midwives, can lead to an improvement in breastfeeding results.

Likewise, we observed that the second most important influence reported by women was the role of the midwife. The importance of midwives in relation to breastfeeding is undeniable [29, 30] and is also closely related with maternal education, a task that falls under the competence of the midwife and which has been proven to be fundamental in increasing breastfeeding rates [10, 21].

The analysis of the reasons for breastfeeding has shown that the main reasons are related with perceiving breastfeeding as the best option for the baby, which is supported by other studies such as Newby [31]. In the study by Bernie [32] even mothers who did not decide to breastfeed recognised the benefits it has for babies’ health.

### Limitations

Several limitations were identified in this study. Firstly, there could be a selection bias due to the lack of participation of women who decided not to breastfeed. For this reason, we believe that although this study is not valid to determine the overall prevalence of the intention to breastfeed, it is adequate to determine the associated factors. It is unlikely that there is an information bias because the data compiled and the way in which the possible responses were presented, did not require a high level of education. In this regard, the questions were formulated in a basic and simple way so that all participants could understand them regardless of their level of education. In the sample, women under the age of 18 were not included, so the results cannot be extrapolated to this population section. It is possible that the extended time period used for data collection may have influenced some of the analysed factors. However, no relevant changes had occurred during this period in pregnancy management or birth attendance in Spain that could have significantly influenced the context. Finally, it is not possible to totally rule out confusion bias inherent to observational studies, although we did attempt to control this using multivariate analysis techniques.

## Conclusions

In conclusion, we can say that in this study, five factors were identified related with the mother’s prenatal decision to breastfeed: partner support, previous experience of breastfeeding, having two or more children, attending breastfeeding education and having a condition during pregnancy. Given the observed influence that partners have when it comes to decision-making, their active participation during the process is enhanced here, both at individual consultations and at maternal education classes. Furthermore, midwives would also be recommended to conduct more outreach with women over the internet and social media, mainly for two reasons: to reach a greater number of women regarding different aspects of health and to consolidate the credibility of midwives as a reliable and rigorous source of information.

## Supplementary Information

**Additional file 1.** List of associations that have collaborated in the distribution of the questionnaire.

## Data Availability

Not applicable.
